# Monoclonal Antibodies Targeting Surface-Exposed and Secreted Proteins from Staphylococci

**DOI:** 10.3390/vaccines9050459

**Published:** 2021-05-04

**Authors:** Pietro Speziale, Giampiero Pietrocola

**Affiliations:** Department of Molecular Medicine, Unit of Biochemistry, University of Pavia, 27100 Pavia, Italy; giampiero.pietrocola@unipv.it

**Keywords:** *Staphylococcus aureus*, *Staphylococcus epidermidis*, virulence factor, infectious diseases, monoclonal antibody, passive immunization

## Abstract

Staphylococci (specifically *Staphylococcus aureus* and *Staphylococcus epidermidis*) are the causative agents of diseases ranging from superficial skin and soft tissue infections to severe conditions such as fatal pneumonia, bacteremia, sepsis and endocarditis. The widespread and indiscriminate use of antibiotics has led to serious problems of resistance to staphylococcal disease and has generated a renewed interest in alternative therapeutic agents such as vaccines and antibodies. Staphylococci express a large repertoire of surface and secreted virulence factors, which provide mechanisms (adhesion, invasion and biofilm development among others) for both bacterial survival in the host and evasion from innate and adaptive immunity. Consequently, the development of antibodies that target specific antigens would provide an effective protective strategy against staphylococcal infections. In this review, we report an update on efforts to develop anti-staphylococci monoclonal antibodies (and their derivatives: minibodies, antibody–antibiotic conjugates) and the mechanism by which such antibodies can help fight infections. We also provide an overview of mAbs used in clinical trials and highlight their therapeutic potential in various infectious contexts.

## 1. Introduction

Staphylococci are commensal bacteria that make up a large part of the microbiota of human and animal tissues. Of the almost 50 species of staphylococci identified so far, most are coagulase-negative, like *Staphylococcus epidermidis*, while a few, such as *Staphylococcus aureus*, are coagulase-positive. *S. epidermidis* is a colonizer of the human skin and nares and plays an important role in the maintenance of healthy skin flora [1,2]. *S. epidermidis* is also recognized as an opportunistic pathogen that can cause important problems, especially during biofilm formation on the surface of indwelling medical devices [3]. *S. aureus* is much more pathogenic and persistently colonizes about 20–30% of the human population. From the primary habitat, the anterior nares can be disseminated to the skin, intestine and deeper host tissues, such as bone and cardiac valves. In pathogenetic conditions, *S. aureus* causes opportunistic and life-threatening infections such as pneumonia, bone and joint infections, endocarditis and sepsis [4]. *S. aureus* expresses a broad range of cell-wall-anchored (CWA) proteins, mostly involved in adhesion to extracellular matrix and plasma proteins [5,6] and lipoproteins involved in different metabolic processes [7]. Moreover, *S. aureus* expresses a multitude of secreted proteins/peptides that compromise innate immune responses [8]. *S. epidermidis* is endowed with a more limited repertoire of virulence factors than *S. aureus*. It can express CWA proteins that interact with extracellular matrix proteins but lacks cytolytic toxins that are produced in abundance by *S. aureus* [2]. Both species use several CWA proteins and the polysaccharide poly-N-acetyl glucosamine (PNAG) for promoting biofilm formation [9,10,11]. Due to the pivotal role they play in bacteria pathogenesis and their prompt accessibility, surface-exposed proteins and secreted virulence factors represent the ideal target for the host immune system. Here we present virulence factors that are involved in tissue colonization and infection by these bacteria, describe their immunological properties and provide an update on monoclonal antibodies against these factors, as well as their potential use in preclinical and clinical trials.

## 2. *Staphylococcus* Virulence Factors

### 2.1. CWA Proteins

**Figure 1:**
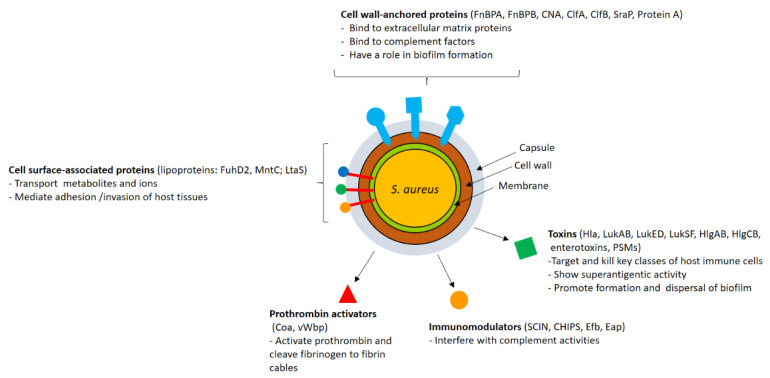
Schematic representation of the *Staphylococcus aureus* surface and secreted virulence factors*. S. aureus* is endowed with a large arsenal of surface-expressed and secreted virulence factors that are targeted by the host immune system. These targets include (a) cell-wall-anchored proteins that promote adhesion to extracellular matrix proteins, binding to complement proteins and biofilm formation; (b) surface-associated proteins anchored to the cell membrane and involved in processes such as transportation of metabolites and ions and enzymatic and ligand-binding activities; and secreted factors, which include leukocidal toxins and superantigens, autolysins, prothrombin activators and modulators of the complement pathways.

**Figure 2:**
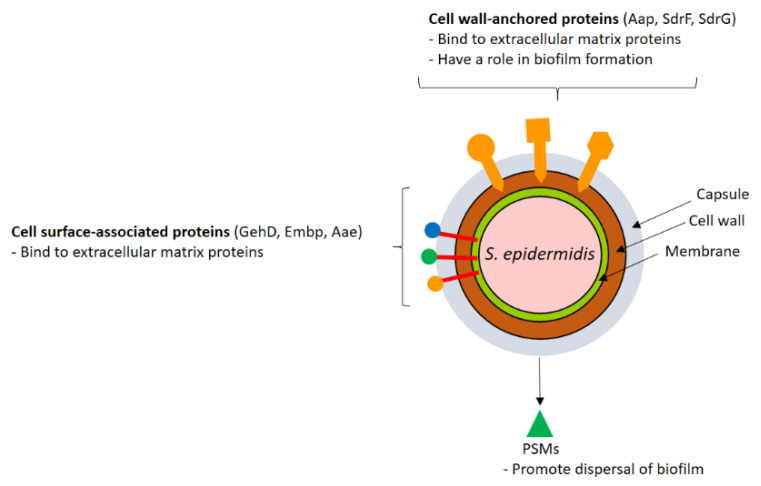
Schematic representation of *Staphylococcus epidermidis* virulence factors. *S. epidermidis* has a smaller repertoire of CWA proteins than *S. aureus*. Cell-wall-anchored and surface-associated proteins interact with extracellular matrix ligands and promote biofilm formation. Secreted proteins include modulators of biofilm disruption such as PSMs.

CWA proteins are covalently anchored to the peptidoglycan scaffold by membrane-associated sortases. Fibronectin-binding proteins A and B (FnBPA and FnBPB) were the first proteins of *S. aureus* to be characterized, and they have been shown to share with clumping factor A (ClfA) the property of binding to fibrinogen with a similar mechanism [12,13]. FnBPA and FnBPB are also involved in plasminogen binding [14], and FnBPB interacts with additional binders, such as histones [15], and corneodesmosin [16]. Moreover, as a result of their fibronectin-binding activity, FnBPA and FnBPB are primarily involved in *S. aureus* colonization of host tissues [17]. A pivotal role in the colonization of tissues, especially nares, is attributed to clumping factor B (ClfB), which shows a multivalent ability to interact with host proteins such as fibrinogen [18,19,20], cytokeratin 10 [21], loricrin [22] and corneodesmosin [16]. CNA, a collagen- and laminin-binding protein [23,24,25], has proven to be a virulence factor in several infectious diseases such as endocarditis [26], osteomyelitis [27], keratitis [28] and arthritis [29]. CNA also binds to C1q and inhibits the classical complement pathway [30]. Staphylococcal protein A (SpA) is another CWA protein that interferes with the action of the innate immune system. SpA has the ability to bind with high affinity to the Fc region of immunoglobulin G (IgG), and this binding on the surface of bacterial cells results in an incorrect orientation, compromising IgG recognition by the Fc receptor on phagocytes. SpA also binds to the VH3 heavy chains of IgM displayed on B lymphocytes, and this interaction triggers the proliferation and depletion of B-cell repertoire and the collapse of the adaptive immune response [31]. Moreover, SpA binds to several other ligands such as von Willebrand factor (vWF) [32] and tumor necrosis factor receptor-I [33]. Through the binding to vWF, SpA mediates staphylococcal adhesion to endothelial cells [34]. The serine-rich adhesin of platelets (SraP), also named SasA, is a CWA glycoprotein that binds N-acetyl neuraminic acid (Neu5Ac) containing glycoproteins, including the salivary glycoprotein gp340 [35,36]. *S. aureus* can also invade a variety of non-professional phagocytic cells such as epithelial and endothelial cells, explaining its capacity to persist in tissues after bacteremia and resist antibiotic treatment and antibody attack. The molecular mechanism by which bacteria invade these cells mainly involves FnBPs. Specifically, bacteria expressing FnBPs interact with the extracellular matrix fibronectin, which, in turn, binds to integrin α5β1 on the surface of the host cells, and the formation of the ternary complex elicits the uptake of bacteria [17]. The iron-regulated surface determinant B (IsdB) protein, primarily involved in the acquisition of iron from host hemoglobin [37], also promotes bacterial binding to the extracellular matrix protein vitronectin and invasion of human cells [38]. Lastly, SraP promotes bacterial adhesion to and invasion of mammalian cells by an unknown mechanism [39] (Figure 1). *S. epidermidis*, the most important coagulase-negative staphylococcal species, has a smaller repertoire of CWA proteins than *S. aureus* [2]. As reported for *S. aureus*, CWA proteins of *S. epidermidis* have the potential to engage extracellular matrix ligands such as collagen, fibrinogen and fibronectin and promote adhesion to the tissues. The most well-known CWA proteins of *S. epidermids* are SdrG/Fbe, which binds fibrinogen [39]; Aap, which plays an important role in biofilm formation [40,41]; and SdrF, involved in adhesion to collagen deposited on ex vivo drivelines [42]. The CWA protein SesC, which reveals fibrinogen-binding activity [43], has been suggested to play a role in biofilm formation [44] (Figure 2).

### 2.2. Lipoproteins and Other Surface-Associated Proteins

*S. aureus* expresses more than 60 surface-exposed lipoproteins (Lpp), which are involved in a variety of metabolic processes such as the uptake of nutrients and ions and enzymatic activities [7]. All Lpp contain a LipoBox motif that allows the protein to be covalently bound to the outer surface of the cytoplasmic membrane through a thioether linkage [45]. Of the variety of lipoproteins identified so far, the ferric hydroxamate-binding Lpp named FhuD2 (ferric hydroxamate uptake D2) [46] and the manganese transporter C (MntC) Lpp [47] have been investigated to establish their role in bacterial fitness, virulence and vaccinal potential. A special class of *S. aureus* Lpp called “lipoprotein-like” lipoproteins (Lpl) contributes to host cell invasion [48]. LtaS is an *S. aureus* membrane-embedded enzyme with five N-terminal transmembrane helices connected via a linker region to the C-terminal extracellular enzymatic domain (eLtaS) involved in lipoteichoic acid synthesis. The soluble eLtaS domain, produced through the cleavage of LtaS by SpsB protease and consequent release from the bacterial membrane, binds to insulin with high affinity. It has been suggested that this binding plays a role in insulin resistance and the potential development of diabetes [49] (Figure 1). *S. epidermidis* expresses the surface-associated autolysin Aae, which promotes adhesion to immobilized plasma proteins vitronectin, fibronectin and fibrinogen, and it is suggested that it is involved in the colonization of animal tissues [50]. Additional *S. epidermidis* surface-associated adhesins are the glycerol ester hydrolase (GehD), which can bind to collagen in vitro [51], and the extracellular matrix binding protein (Embp) involved in *S. epidermidis* interaction with fibronectin [52,53] (Figure 2).

### 2.3. Secreted Proteins

#### 2.3.1. Factors Interfering with the Host Immune System

*S. aureus* secretes a wide range of proteins/peptides that target different parts of the host defense system. Staphylococcal complement inhibitor (SCIN) binds to C3 convertases of the classical, lectin and alternative pathways, and this results in a decline of C3b formation/deposition and reduced production and release of chemoattracting peptide C5a. Consequently, SCIN blocks phagocytosis and the killing of *S. aureus* cells by the phagocytic cells [54]. *S. aureus* impairs the response of neutrophils and monocytes to formylated peptides and C5a using the specific secreted peptide CHIPS (chemotaxis inhibitory protein of *Staphylococcus aureus*). CHIPS binds the formyl peptide receptor and the C5a receptor, and binding to these receptors reduces neutrophil activation and migration to the site of infection [55,56]. The staphylococcal extracellular fibrinogen-binding protein Efb and its homolog extracellular complement binding protein (Ecb) block C3 and C5 convertases of the alternative pathway, thereby lowering opsonization and recruitment of neutrophils [57]. Efb can also inhibit the interaction of fibrinogen with neutrophils in vitro, thus preventing neutrophil transmigration and/or fibrin-supported neutrophil activation [58]. The multifunctional extracellular adherence protein (Eap) potently inhibits formation of the classical and lectin C3 proconvertase (C4bC2) by interfering with the binding of both full-length C2 and C2b fragment to C4b. Consequently, Eap inhibits the deposition of C3b onto the surface of *S. aureus* cells and reduces the level of *S. aureus* opsonophagocytosis and the killing by neutrophils [59]. Furthermore, Eap specifically inhibits the activity of neutrophil proteases involved in immune defense [60]. *S. aureus* secretes two hemostasis factors, coagulase (Coa) [61,62] and von Willebrand factor binding protein (vWbp) [63], which bind to and activate prothrombin in a non-proteolytic manner [62]. Coa-prothrombin and vWFbp-prothrombin complexes (staphylothrombin) share the ability to cleave fibrinogen and form fibrin cables that protect the pathogen against phagocytosis by immune cells [64,65]. Both the proteins bind to fibrinogen [66,67], and vWFbp also interacts with vWF [62,68] and fibronectin [66].

#### 2.3.2. Toxins

To further contribute to pathogenesis, *S. aureus* secretes the cytolytic toxin Hla, leukocidins and enterotoxins. Hla is a toxin secreted as a monomer that, upon binding to its receptor, the Zn^2+^-dependent metalloprotease ADAM-10, forms homo-heptameric pores in host cell membranes. Hla elicits ADAM10-mediated cleavage of endothelial tight junctions and alteration of vascular integrity. It also contributes to *S. aureus* pathogenesis, preventing repair of the injured endothelial barrier and causing dysfunctions of organs through plateletneutrophil aggregate formation [69]. Five leukocidins have been described in human-associated strains of *S. aureus*, γ hemolysins HlgAB and HlgCB, PantonValentine leukocidin (PVL or LukSF), LukED and LukGH (also known as LukAB). Leukocidins consist of two independent secreted monomers, termed S- and F-subunits, which assemble to form octameric pores in the plasma membranes and elicit lytic activity toward human phagocytic cells [69]. Enterotoxins are a family of over 20 secreted toxins with superantigenic activity. The enterotoxins with the most powerful superantigenic activity are the staphylococcal enterotoxin B (SEB) and toxic shock syndrome toxin-1 (TSST-1) [69]. At the molecular level, SEB and TSST-1 form a bridge between the major histocompatibility complex class II molecules on antigen-presenting cells and Vβ chains of T-cell receptors (TCRs), resulting in a massive release of pro-inflammatory cytokines and a fatal condition known as toxic shock syndrome (TSS). TSS is an acute systemic illness characterized by hypotension, fever and rash. While *S. aureus* expresses a vast repertoire of toxins, *S. epidermidis* is not considered an enterotoxin producer, and its toxin production is limited to phenol-soluble modulins (PSMs), which are amphipathic peptides involved in several activities, including the remodeling and dispersion of staphylococcal biofilm [1] (Figure 1 and Figure 2).

### 2.4. Quorum Sensing Regulatory System

Quorum sensing (QS) is the mechanism by which staphylococci communicate with each other via diffusible molecules and adjust gene expression accordingly. *uorum sensing is a mechanismS. aureus* uses a canonical Gram-positive quorum sensing system encoded by the *agr* (accessory gene regulation) locus. A central role in the *S. aureus agr* QS system is played by a secreted thiolactone-cyclized autoinducing 7–9 residue peptide (AIP) which is synthesized from the 45–47 residue AgrD precursor. The AIP endopeptidase AgrB processes AgrD to mature AIP and transports it out of the cells. When AIP accumulates in the extracellular milieu at a level as high as 10 M, it binds the transmembrane sensor histidine kinase AgrC, which activates and self-phosphorylates to a conserved histidine and transfers the phosphate group to an aspartate on the response regulator AgrA. Phosphorylated AgrA activates a regulatory cascade leading to changes in the transcription of more than 200 genes. The upregulated genes include those encoding secreted virulence factors such as Hla and exoenzymes, while genes for the expression of FnBPA and FnBPB, protein A, coagulase and other surface proteins are repressed. Interestingly, *S. aureus* strains can be divided into four distinct *agr* subgroups, generally referred to as groups I, II, III and IV. In each *agr* group, the AgrC receptor recognizes a specific AIP structure (i.e., AIP-1 through AIP-4). Blockage of quorum sensing has been shown to attenuate the expression of virulence factors in Gram-positive bacteria [10].

### 2.5. Biofilm Formation

Biofilms are multicellular microbial communities encased within a self-produced extracellular polymeric substance comprising polysaccharides such as PNAG, proteins and extracellular DNA (eDNA). Biofilm infections are clinically important because bacteria in biofilms afford increased resistance to antimicrobial compounds and host immunity [9,10,11]. Treatment of biofilm infection is complicated by the fact that often it occurs in areas of the body that are poorly accessible for therapeutic treatment. Importantly, biofilm development by staphylococci is often associated with medical implants such as prosthetic joints, catheters and heart valves [3]. The development of a bacterial biofilm is a complex, multifactorial process and can be divided into three phases: attachment, maturation and detachment. Several CWA proteins from *S. aureus* (ClfA, ClfB, SasG, SdrC, FnBPA/FnBPB) and Aap from *S. epidermidis* [9] play a role in biofilm formation. Secreted proteins such as Hla [70,71] and LukAB [72] and specific DNA-binding proteins (DNABII) [73,74,75], which stabilize the eDNA in biofilm matrix, have been also implicated in biofilm development. The molecular mechanism by which CWA proteins contribute to biofilm formation is well established, while the involvement of secreted proteins in biofilm development is still unclear.

## 3. Antibodies

### 3.1. General Properties of Antibodies

Antibodies are proteins made up of two heavy and two light chains, and each chain has both a variable and a constant region. An antibody can be cleaved by papain into three pieces, two identical Fab fragments and one Fc fragment. Each Fab fragment contains the variable region that binds the antigen, while the Fc fragment consists of the constant region that interacts with cell surface Fc receptors and proteins of the complement system, such as C1q. Although all antibodies have the same overall structure, each antibody has a unique antigen-binding site that enables it to bind to its corresponding epitope on the antigen. Antibodies are produced by the B lymphocytes in response to infection or immunization with an antigen and bind to and neutralize pathogens and prepare them for uptake and destruction by phagocytes. Among the five classes of antibodies, IgGs are the most abundant ones in serum (80–85% of the total immunoglobulins in serum). The IgGs can be subdivided into four subclasses (IgG1, IgG2, IgG3 and IgG4) and are numbered in accordance with their decreasing average in the serum, with IgG1 being the most abundant. The properties that distinguish the subclasses from one to another include the molecular weight, the serum half-life and the hinge length. IgG1, IgG3 and IgG4 readily cross the placenta and play an important role in protecting the developing fetus. The IgG3 subclass is the most effective classical pathway complement activator, followed by IgG1, while IgG2 and IgG3 are relatively ineffective. IgG3 and IgG1 bind with high affinity to Fc receptor of macrophages and other phagocytic cells, whereas IgG4 and IgG2 have a low affinity for it [76,77].

### 3.2. Monoclonal Antibodies and Their Derivatives

Monoclonal antibodies (mAbs) are produced by a single clone of B lymphocytes and are usually generated in the laboratory by making hybrid antibody-forming cells from a fusion of non-secreting myeloma cells with immune spleen cells. Since the development of hybridoma technology, molecular cloning and recombinant expression methods have provided tools for “reshaping” the antibody molecule to obtain antibody fragments (minibodies or nanobodies), humanized or fully human antibodies. Examples of minibodies are single-chain variable-fragment (scFv) antibodies which consist of only the light chain and heavy chain variable regions of immunoglobulins connected by a peptide linker. ScFv antibodies contain the antigen-binding site and are as specific and affine as intact antibodies. Humanized antibodies are structural chimeras resulting from the fusion of the Fc region originating from one species, usually human, with the variable region from another species, usually murine. This allows a chimeric antibody to interact with the human immune system and minimize the risk of an adverse reaction by the human immune system to the antibody administered [78].

### 3.3. Active and Passive Immunization

Vaccination (active immunization) is the deliberate induction of adaptive immunity to a pathogen by injecting a vaccine, which is a dead or attenuated form of the pathogen or a toxin. Transfer of immunity by antiserum or purified exogenous antibodies such mAbs (passive immunization) provides protection against many pathogens or toxins. When used for therapeutic purposes, a mAb must be selected for the antigen affinity, subclass to which it belongs, the potential ability to neutralize the biological activity of the corresponding antigen and the absence of cross-reactivity with other antigenic components of the host. Unlike vaccination, which raises a specific immune response and offers lasting protection after the administration of a specific antigen, the administration of antibodies provides immediate but temporary protection to recipients who have been exposed to pathogens. This could be beneficial for subjects with immune deficiencies and in emergency conditions.

## 4. mAbs Targeting Virulence Factors and Their Possible Therapeutic Use to Combat Staphylococcal Infections

Antibodies have proven to be effective in the treatment of cancer and inflammatory diseases and in the neutralization of pathogens. The initial formulation of antibody therapy against infections was based on the use of immune sera and, later, on the use of immunoglobulin preparations made of purified IgG from which viruses and impurities have been removed. The discovery and production of mAbs quickly replaced the use of heterologous sera. In contrast to polyclonal antibodies, the use of mAb therapy has several advantages including (i) little cross-reactivity with both host cells and normal bacterial flora, (ii) higher potency of the mAbs per mass of protein used and (iii) low chemical variability and high specificity. Over the past decade several mAbs against surface and secreted proteins from staphylococci have been shown to be effective both in vitro and in animal infection models (Table 1).

### 4.1. mAbs against Surface and Surface-Associated Proteins

mAbs to combat different staphylococcal surface antigens have been developed or are being developed at the research level. Early production and characterization of anti-*S. aureus* mAbs against the collagen/laminin-binding adhesion CNA paved the way for the generation of mAbs against other CWA proteins of *S. aureus*. The members of the anti-CNA antibody family recognize conformational epitopes and are able to block the attachment of *S. aureus* cells to collagen/laminin substrates and effectively displace bacteria that previously adhered to collagen. They also block the binding of C1q to CNA [83,84]. The mouse mAb Aurexis and its humanized version tefibazumab, which target ClfA, resulted effective in animal models when used in combination with conventional antibiotics, but they exhibited limited action in a phase II clinical trial (Table 2) [79,80,81]. A family of murine mAbs against the fibrinogen-binding protein SdrG from *S. epidermidis* has been generated and characterized, but their efficacy as therapeutic agents in animal models is still to be determined [99]. Schaffer et al. generated an anti-ClfB monoclonal antibody that inhibits adhesion of *S. aureus* to surface-coated cytokeratin 10 and showed that passive immunization with this antibody significantly reduced nasal colonization compared with the colonization of nares observed when the animals were immunized with an isotype-matched unrelated antibody [82]. Murine mAbs against IsdB were shown to enhance the opsonophagocytic killing of *S. aureus* cells and to induce protection in lethal challenge models and a sublethal indwelling catheter model [100].

mAbs against a non-toxigenic mutated isoform of SpA isolated by Kim et al. were shown to prevent the binding of immunoglobulins to SpA and neutralized the Fc- and Fab-binding activities of SpA. Anti-SpA mAbs also promoted the opsonophagocytic killing of methicillin resistant *S. aureus* (MRSA) in human and mouse blood and provided protection from abscess formation [85]. The mouse mAb 3F6 binds to each of the five immunoglobulin-binding domains (IgBDs) of SpA, blocks IgG binding to SpA and protects against bloodstream infection when administered to mice [85,86,87]. A human version of this antibody (3F6-hIgG1) improves the outcome of MRSA bloodstream infections in animal models [87,88] and its Fc glycosylation enhances C1q binding and the recruitment and killing of staphylococci by phagocytosis and favors protection against infection [101]. Moreover, Varshney et al. isolated and studied a human mAb, named 514G3, belonging to the subclass IgG3, a condition that does not allow binding via the Fc region to SpA. 514G3 bound with high-affinity SpA via its CDRs supports phagocytosis and the killing of *S. aureus* by human blood cells and protects mice against lethal challenge with MRSA in a bacteremia model. Notably, sub-efficacious doses of antibody combined with suboptimal doses of vancomycin, a standard antibiotic used to treat MRSA bacteremia, successfully showed prophylactic efficacy in mice upon MRSA challenge [89]. A phase I study of 514G3 antibody in patients hospitalized with *S. aureus* bacteremia has been reported [102] (Table 2).

Finally, a mAb named 2H7 raised against the large surface protein SraP (SasA) from *S. aureus*, when passively administered to mice, promoted the survival of animals challenged with bacteria and significantly enhanced clearance in kidneys in both sepsis and peritoneal infection. Prophylactic administration of the antibody also reduced the formation of intraperitoneal abscess in mice with peritoneal infection and induced protection in a murine sepsis model [103]. Moreover, a monoclonal antibody raised against L-lectin domain of protein SraP inhibited attachment of bacteria to the A549 epithelial cell line, and passive immunization with the mAb significantly reduced the number of bacteria in the blood [90].

A family of mAbs to lipoprotein MntC generated by Anderson et al. bound to *S. aureus* and *S. epidermidis* cells, creating protection in an infant rat immunoprophylaxis model and facilitating respiratory burst activity of neutrophils [104]. The single-chain mAb fragment Aurograb, which targets the *S. aureus* ATP-binding cassette (ABC) transporter GrfA, worked synergistically with vancomycin to reduce infection in mice by *S. aureus*, but it proved ineffective in a Phase II trial [105,106,107] (Table 2). Finally, Liu et al. developed a human mAb against the C-terminal extracellular fragment (eLtaS) of the membrane-associated enzyme LtaS that inhibits the interaction of the protein with insulin [49]. It remains to be seen whether the effects of colonization and chronic *S. aureus* infections could be managed by employing the function-blocking eLtaS antibody, possibly in conjunction with antimicrobial therapy. This study provides a link between *S. aureus* infection and the potential development of diabetes in humans and prospectively opens a new avenue for therapeutic intervention in the treatment of S. aureus-induced insulin resistance.

### 4.2. mAbs against Secreted Proteins/Peptides

A number of mAbs that target secreted virulence factors and their therapeutic validation in animals are under development. The C-terminal domain or R domain of Coa binds to fibrinogen and consequently generates a protective fibrin shield that blocks phagocytosis. Binding of the mAb 3B3 directed to the R domain of Coa blocks the above process, triggering phagocytosis and the killing of staphylococci, and protects mice against lethal bloodstream infections caused by MRSA isolates [91].

Georgoutsou-Spyridonos et al. also developed recombinant mini-antibodies that neutralize the function of Efb by blocking its interaction with complement C3 both in vitro and in vivo. The prophylactic use of these mini-antibodies potently attenuated the survival of *S. aureus* in a whole-blood model of bacteremia and attenuated bacterial inflammation in the kidney in a murine renal abscess model [108]. mAbs were also raised against the protein CHIPS. The biological effects of the components of this family of mAbs were different. Some mAbs only blocked the binding of CHPS to formyl peptide receptor, while others prevented CHIPS from binding to C5a receptor without blocking interaction with formyl peptide receptor. A mAb that interfered with CHIPS activity on both receptors was also identified. Further studies are needed to establish the therapeutic values of these antibodies in vivo [109]. A human mAb named 6D4 was obtained by Hoekstra et al. from the random screening of B lymphocytes that bind to whole *S. aureus* cells. 6D4 inhibited SCIN activity, as demonstrated by the analysis of C3b deposition on *S. aureus* cells. However, it is unlikely that 6D4 can be used in anti-staphylococcal therapy, as SCIN-deficient mutants of *S. aureus* continue to express their pathogenetic potential [110]. A human mAb against Hla termed MEDI4893 developed by Medimmune blocks the Hla region from binding to cellular receptor ADAM10 and inhibits pore formation. MEDI4893 affords protection to mice challenged with *S. aureus* in a model of acute pneumonia [94,95]. Safety, tolerability and pharmacokinetics of MEDI4893 mAb and its immunoprophylactic effects against *S. aureus* disease in human subjects have been reported [96,97] (Table 2).

Arsanis produced cross-reactive, high-affinity antibodies for Hla and four different bi-component leukocidins (HlgAB, HlgCB, LukED and LukSF). A single human mAb with these properties prevented the lysis of phagocytes, epithelial cells and erythrocytes determined by the toxins in vitro and elicited high-level protection in murine models of sepsis and pneumonia [111,112]. Clinical trials, however, failed (Table 2). Treatment with humanized SEB-neutralizing 20B1 protected mice both in sepsis and in a SEB intoxication model [113].

Along this line, passive therapy with human–mouse chimeric, high-affinity anti-SEB antibodies mitigated the systemic inflammatory response that occurs during pneumonia caused by SEB-producing *S. aureus*. The same antibodies administered in combination with linezolid afforded protection from lethal pneumonia [114]. Prophylactic treatment with a combination of two mAbs, named 4G3 and 5G4, that recognize different epitopes on staphylococcal enterotoxin K (SEK) significantly enhanced the survival of mice in a model of SEK-induced lethal shock and sepsis by a virulent staphylococcal strain [98] (Table 1). Human monoclonal single-chain antibodies (scFvs) against TSST-1 blocked the activation and proliferation of T cells and the secretion of pro-inflammatory cytokines, suggesting their possible therapeutic application in infective syndromes involving this important superantigen [115].

### 4.3. mAbs That Block the Agr QS System

As reported above, in many pathogens, quorum-sensing systems control the expression of virulence factors. Quorum quenching has often been proposed as a bacterial strategy that broadly affects and neutralizes bacterial virulence. Park et al. described the production of a mAb named AP4-24H11 that binds with nM affinity to AIP-4 used by *S. aureus agr* group IV strains as a QS signaling molecule [92]. The crystal structure of AP4-24H11 antibody in combination with AIP4 reveals that the antibody recognizes the characteristic AIP thiolactone ring conformation [93]. Treatment of *S. aureus* cultures with AP4-24H11 antibody increased protein A expression and reduced Hla expression and RNA III transcription, in accordance with the suppression of QS signaling. Moreover, passive transfer of AP4-24H11 afforded protection against *S. aureus* pathogenicity in a mouse model of dermonecrosis and against a lethal intraperitoneal *S. aureus* challenge. However, we must bear in mind that Agr quorum sensing downregulates the production of PSMs, the staphylococcal peptides that trigger biofilm remodeling. Consequently, inhibition of PSMs production may result in the formation of thick, undifferentiated biofilm. Caution must be exercised when using anti-QS antibodies as an anti-biofilm strategy.

### 4.4. mAbs That Inhibit Biofilm Development

Consistent with the important role played by Aap in biofilm formation, mAbs targeting this surface protein inhibit *S. epidermidis* biofilm formation [116]. Estellès et al. produced a human mAb named TRL1068 that cross-reacts with a DNABII epitope shared by several Gram-positive and Gram-negative species and showed that the antibody effectively disrupts the staphylococcal biofilm in vitro as well as the catheter-associated biofilm in a rat model of infection [73]. Due to this effect on biofilm dispersal, TRL1068 has been proposed as a candidate for the treatment of implant-associated infections in combination with antibiotics [73]. Hla promotes biofilm formation and is required for cell-to-cell interactions during biofilm formation [70,71]. The above-reported human monoclonal antibody anti-Hla MED14893 successfully abrogated ex vivo biofilm formation on porcine vaginal mucosa explants [71]. Moreover, prophylactic treatment with MEDI4893 promotes wound healing in a mouse model of *S. aureus* wound infection, suggesting that this antibody may be used for the treatment of biofilm-related wound infections [94,117]. Furthermore, the combination of human monoclonal antibodies against Hla and ClfA strongly inhibited biofilm formation in vitro and hematogenous implant-related infection in vivo [118].

## 5. Antibody-Conjugated Derivatives and Their Use to Combat Staphylococcal Infections

mAbs generated against *S. aureus* antigens have been profitably used for the generation of promising mAb-based derivatives. In one such effort, a drug consisting of a specific antibody targeting the wall teichoic acid of *S. aureus* and a potent covalently conjugated antibiotic belonging to the rifamycin class, when used to opsonize bacteria, was shown to efficiently eradicate intracellular *S. aureus* cells trapped inside murine macrophages. A critical point of this action includes the enzymatic cleavage of the linker connecting the antibody to the bioactive payload and the consequent release of the antibiotic within the macrophages. Parallel studies performed in vivo have shown that this therapeutic platform efficiently reduces the bacterial load in the kidneys, heart and bone [119,120]. This innovative approach offers an exciting opportunity to set up derivatives where components of the therapeutic platform (mAb, antibiotic and linker) are selected according to specific needs such as the lifestyle of the infecting strain (planktonic vs. sessile), type of infection (systemic vs. local), bacterial repertoire of virulence factors, pathogen-specific antibiotic and specific structure of the linker.

Bispo et al. used a targeted antimicrobial photodynamic therapy (aPDT) consisting of the monoclonal antibody 1D9 specific for the invariantly expressed immunodominant staphylococcal antigen A (IsaA) conjugated with a photosensitizer named IRDye700DX. The resulting immunoconjugate 1D9-IRDye700DX, when activated by illumination with red light, induced generation of ROS and the consequent disruption of *S. aureus* biofilms in vitro and rescued larvae of the wax moth *Galleria mellonella* from staphylococcal infections. Moreover, the immunoconjugate proved effective in the killing of *S. aureus* in a human postmortem infection model [121]. Likewise, gold nanoparticles conjugated to monoclonal antibodies specific to *S. aureus* peptidoglycan, when incubated with suspensions of methicillin-resistant and methicillin-sensitive *S. aureus* and activated by pulsed laser irradiation, significantly reduced bacterial viability thanks to photothermal killing [122]. New antibody treatment could be used either alone or combined with conventional antimicrobial therapy. Lastly, antibody-based biologicals could be used to diagnose biofilm formation in the body. Recently, de Vor. et al. reported the successful use of ^111^In-labeled mAb 4497 which recognizes wall teichoic acid (WTA) to localize biofilm, in a mouse implant infection model [123]. These data may be the prelude to the use of anti-staphylococcal mAbs labeled with a radionuclide for early diagnosis of biofilm-related infection in patients with implanted medical devices and for detection of biofilms during review surgery or in explanted implants.

## 6. Discussion and Conclusions

In this review, we have described the variety of staphylococcal virulence factors, mainly surface and secreted proteins, and highlighted the importance of these factors in the onset of staphylococcal disease. We have also reported on the generation of mAbs against these antigens and their successful use to inhibit the agent activity in vitro and in animal models. Despite considerable efforts aimed at the generation and testing of mAbs against staphylococcal antigens in passive immunization studies, so far, the use of humanized and human mAbs has not been decisive in mitigating the severity of bacterial infections in preclinical and clinical trials. There are several reasons for this failure. Primarily, considering the huge variety and redundancy of staphylococcal virulence factors, the use of antibodies targeting a single antigen in passive immunization might compromise the success of the treatment. Second, the impact of IgG-binding proteins expressed by *S. aureus* (protein A, Sbi) and other immune evasion factors (SCIN, CHPS, Efb) has to be considered. Third, due to the different genetic and immunological backgrounds, the protection data obtained with animal models cannot be simply extrapolated to preclinical/clinical trials in humans. For example, SCIN and its homologs strongly inhibit alternative pathway-mediated hemolysis in human serum, while no inhibition was observed in mice and other species tested [57]. In view of these considerations, the production of mAbs against mutated isoforms of SpA that neutralize Fc and Fab binding activities and block IgG binding to SpA may add value to therapeutic strategies based on the use of other anti-staphylococcal mAbs. Therefore, one should bear in mind that a successful prophylactic treatment should be based on the use of a cocktail of mAbs targeting different staphylococcal antigens where the presence of mAbs neutralizing Fc and Fab binding activities of SpA is essential.

When mAbs are used for therapeutic purposes in humans, additional issues need to be taken into due consideration. MAbs against a bacterial antigen could cross-react with patient tissue components, and the formed immune complexes could trigger adverse side effects in the host. As an example, mAbs against specific antigens of group A streptococci show cross-reactivity with normal and psoriatic human skin components [124]. Furthermore, mAbs could induce untoward immunotoxic effects such as immunosuppression or immunostimulation of the host immune system or show a direct immunogenic activity [125]. So far, clinical studies related to immunotoxicity of mAbs used for treating bacterial (staphylococcal) infections have been inadequate. Thus, with the ongoing use of mAbs as therapeutic tools against staphylococcal diseases, more efforts should be made to improve our understanding of cross-reactivity and mAb-induced immunotoxic effects and to design mAbs exhibiting immunological safety.

Besides the use of mAbs in passive immunization, the development of antibody-conjugated antibacterial agents may have beneficial effects against staphylococcal infections. For example, the development of antibodyantibiotic conjugates proved highly effective in combating bacteria restricted to intracellular compartments [120,121]. The application of this platform might be potentially extended to staphylococcal biofilm or to bacteria localized in specific niches of the body where the binding of an antibody to a particular antigen concentrates the antibiotic and increases its effectiveness. New technologies are also emerging where light-absorbing gold particles have been functionalized with specific mAbs to achieve targeted and selective laser bacterial killing [122]. In conclusion, present and future knowledge of the pathophysiology of *S. aureus/S. epidermidis* and lessons learned from preclinical and clinical studies represent the basis for the effective use of mAbs and mAb-based biologicals to prevent and treat diseases caused by these remarkable bacteria.

## Figures and Tables

**Table 1:** Targets for antibody-based therapies against *S. aureus*.

Antigen (UniProtKB Accession Code)	Antibody	Ig Class	Recognized Domain/Epitope	In Vitro Activities of the Antibody	In Vivo Efficiency of the Antibody	Refs
ClfA (Q5HHM8)	Tefibazumab, a humanized mAb	IgG_1_	N3 subdomain of A region	Blocks fibrinogen binding to ClfA.	Protects against infective endocarditis.	[79,80,81]
ClfB (O86476)	Mu/3D6	IgG_1k_	N2-N3 subregion	Inhibits bacterial binding to cytokeratin 10.	Reduces nasal colonization by bacteria.	[82]
CNA (Q53654)	Mu/mAbs	IgG_1k_	CNA_151-318_	Inhibits collagen binding to bacteria.	–	[83,84]
SpA (A0A0H3K686)	Mu/Hum/3F6 Mu/514G3	3F6: IgG_2a_ 514G3: IgG_3_	3F6: binds to each of the five immunoglobulin binding domains.514G3: binds to epitope by CDR.	3F6: neutralizes domains of SpA associated with IgG/IgM binding. 514G3: induces phagocytosis.	3F6: protects animals against bloodstream infection. 514G3: rescues mice from bacteria-mediated bacteremia.	[85,86,87,88,89]
SraP (Q5HCP3)	Mu/mAb	Unknown	Lectin domain	Inhibits bacterial adherence to epithelial cells.	–	[90]
LtaS (Q5HHV4)	Hum/YG2	Unknown	Extracellular domain of LtaS (eLtaS)	Inhibits the interaction between eLtsA and insulin.	Restores impaired glucose tolerance in mice.	[49]
Coa (P07767)	Mu/Hum/3B3	IgG_1_	C-terminal R domain	Promotes phagocytosis of fibrin-coated bacteria.	Protects mice against lethal bloodstream infection.	[91]
Autoinducing peptide-4 (AIP-4)	Mu/mAb AP4-Z4H11	Unknown	YSTCYFIM	Inhibits QS in vitro through sequestration of AIP-4.	Suppresses bacterial pathogenicity in an abscess formation mouse model.	[92,93]
DNABII (Q5HFV0)	Hum/TRL 1068	IgG_1_	GRNPQTGKEID	Disrupts biofilm formation.	Blocks biofilm formation in a murine tissue cage infection model.	[73]
Hla (P09616)	Hum/MEDI4893	IgG_1k_	Recognizes a conformational epitope in the “rim” domain of Hla.	Blocks the binding to ADAM10.	Affords protection to mice in a model of acute pneumonia.	[94,95,96,97]
SEK (A0A0H2WWN7)	Mu/mAb 4G3 Mu/mAb 5G2	4G3: IgG_2b_ 5G2: IgG_1_	4G3 and 5G2 recognize distinct epitopes on SEK.	4G3 and 5G2 inhibit SEK-induced proliferation of human immune cells.	Combination of 4G3 + 5G2 enhances survival of mice infected with bacteria.	[98]

**Table 2:** Efficacy of mAbs against *S. aureus* in clinical trials.

Agent	Target Antigen	Primary Clinical Indication	Status
Aurexis (tefibazumab)	ClfA	Treatment of bacteremia	Not in active development
Aurograb	GrfA	Treatment of deep-seated infection	Ineffective in a phase II trial
MEDI4893	Hla	Prevention of pneumonia	Phase II ongoing
514G3	SpA	Treatment of bacteremia	Phase I/II ongoing
ASN100	Hla, HlgAB, HlgCB, LukED and LukSF	Treatment of pneumonia	Clinical trial failed

## Data Availability

Not applicable.

## References

[1] Otto M. (2009). Staphylococcus epidermidis—The ‘accidental’ pathogen. Nat. Rev. Microbiol..

[2] Foster T.J. (2020). Surface Proteins of Staphylococcus epidermidis. Front. Microbiol..

[3] Arciola C.R., Campoccia D., Montanaro L. (2018). Implant infections: Adhesion, biofilm formation and immune evasion. Nat. Rev. Microbiol..

[4] Lowy F.D. (1998). Staphylococcus aureusInfections. N. Engl. J. Med..

[5] Foster T.J. (2019). The MSCRAMM Family of Cell-Wall-Anchored Surface Proteins of Gram-Positive Cocci. Trends Microbiol..

[6] Foster T.J. (2019). Surface Proteins of Staphylococcus aureus. Microbiol. Spectr..

[7] Nguyen M.-T., Matsuo M., Niemann S., Herrmann M., Götz F. (2020). Lipoproteins in Gram-Positive Bacteria: Abundance, Function, Fitness. Front. Microbiol..

[8] De Jong N.W.M., Van Kessel K.P.M., Van Strijp J.A.G. (2019). Immune Evasion by Staphylococcus aureus. Microbiol. Spectr..

[9] Speziale P., Pietrocola G., Foster T.J., Geoghegan J.A. (2014). Protein-based biofilm matrices in Staphylococci. Front. Cell. Infect. Microbiol..

[10] Cheung G.Y.C., Bae J.S., Otto M. (2021). Pathogenicity and virulence of Staphylococcus aureus. Virulence.

[11] Nguyen H.T., Nguyen T.H., Otto M. (2020). The staphylococcal exopolysaccharide PI–Biosynthesis and role in biofilm formation, colonization, and infection. Comput. Struct. Biotechnol. J..

[12] Speziale P., Pietrocola G. (2020). The Multivalent Role of Fibronectin-Binding Proteins A and B (FnBPA and FnBPB) of Staphylococcus aureus in Host Infections. Front. Microbiol..

[13] Ganesh V.K., Rivera J.J., Smeds E., Ko Y.-P., Bowden M.G., Wann E.R., Gurusiddappa S., Fitzgerald J.R., Höök M. (2008). A Structural Model of the Staphylococcus aureus ClfA–Fibrinogen Interaction Opens New Avenues for the Design of Anti-Staphylococcal Therapeutics. PLoS Pathog..

[14] Pietrocola G., Nobile G., Gianotti V., Zapotoczna M., Foster T.J., Geoghegan J.A., Speziale P. (2016). Molecular Interactions of Human Plasminogen with Fibronectin-binding Protein B (FnBPB), a Fibrinogen/Fibronectin-binding Protein from Staphylococcus aureus. J. Biol. Chem..

[15] Pietrocola G., Nobile G., Alfeo M.J., Foster T.J., Geoghegan J.A., De Filippis V., Speziale P. (2019). Fibronectin-binding protein B (FnBPB) from Staphylococcus aureus protects against the antimicrobial activity of histones. J. Biol. Chem..

[16] Towell A.M., Feuillie C., Vitry P., Da Costa T.M., Mathelié-Guinlet M., Kezic S., Fleury O.M., McAleer M.A., Dufrêne Y.F., Irvine A.D. (2021). Staphylococcus aureusbinds to the N-terminal region of corneodesmosin to adhere to the stratum corneum in atopic dermatitis. Proc. Natl. Acad. Sci. USA.

[17] Speziale P., Arciola C.R., Pietrocola G. (2019). Fibronectin and Its Role in Human Infective Diseases. Cells.

[18] Walsh E.J., Miajlovic H., Gorkun O.V., Foster T.J. (2008). Identification of the Staphylococcus aureus MSCRAMM clumping factor B (ClfB) binding site in the αC-domain of human fibrinogen. Microbiology.

[19] Perkins S., Walsh E.J., Deivanayagam C.C.S., Narayana S.V.L., Foster T.J., Höök M. (2001). Structural Organization of the Fibrinogen-binding Region of the Clumping Factor B MSCRAMM of Staphylococcus aureus. J. Biol. Chem..

[20] Ganesh V.K., Barbu E.M., Deivanayagam C.C.S., Le B., Anderson A.S., Matsuka Y.V., Lin S.L., Foster T.J., Narayana S.V.L., Höök M. (2011). Structural and Biochemical Characterization of Staphylococcus aureus Clumping Factor B/Ligand Interactions. J. Biol. Chem..

[21] Walsh E.J., O’Brien L.M., Liang X., Hook M., Foster T.J. (2004). Clumping Factor B, a Fibrinogen-binding MSCRAMM (Microbial Surface Components Recognizing Adhesive Matrix Molecules) Adhesin of Staphylococcus aureus, Also Binds to the Tail Region of Type I Cytokeratin 10. J. Biol. Chem..

[22] Mulcahy M.E., Geoghegan J.A., Monk I.R., O’Keeffe K.M., Walsh E.J., Foster T.J., McLoughlin R.M. (2012). Nasal Colonisation by Staphylococcus aureus Depends upon Clumping Factor B Binding to the Squamous Epithelial Cell Envelope Protein Loricrin. PLoS Pathog..

[23] Speziale P., Höök M., Switalski L.M., Wadström T. (1984). Fibronectin binding to a Streptococcus pyogenes strain. J. Bacteriol..

[24] Switalski L.M., Patti J.M., Butcher W., Gristina A.G., Speziale P., Höök M. (1993). A collagen receptor on Staphylococcus aureus strains isolated from patients with septic arthritis mediates adhesion to cartilage. Mol. Microbiol..

[25] Valotteau C., Prystopiuk V., Pietrocola G., Rindi S., Peterle D., De Filippis V., Foster T.J., Speziale P., Dufrene Y.F. (2017). Single-Cell and Single-Molecule Analysis Unravels the Multifunctionality of theStaphylococcus aureusCollagen-Binding Protein Cna. ACS Nano.

[26] Thomas M.G., Peacock S., Daenke S., Berendt A.R., Young B., Johnson S., Minoo B., Shugarts D., Allen M., Ramey R.R. (1999). Adhesion of Staphylococcus aureus to Collagen Is Not a Major Virulence Determinant for Septic Arthritis, Osteomyelitis, or Endocarditis. J. Infect. Dis..

[27] Elasri M., Thomas J., Skinner R., Blevins J., Beenken K., Nelson C., Smelter M. (2002). Staphylococcus aureus collagen adhesin contributes to the pathogenesis of osteomyelitis. Bone.

[28] Rhem M.N., Lech E.M., Patti J.M., McDevitt D., Hook M., Jones D.B., Wilhelmus K.R. (2000). The Collagen-Binding Adhesin Is a Virulence Factor in Staphylococcus aureus Keratitis. Infect. Immun..

[29] Patti J.M., Bremell T., Krajewska-Pietrasik D., Abdelnour A., Tarkowski A., Rydén C., Höök M. (1994). The Staphylococcus aureus collagen adhesin is a virulence determinant in experimental septic arthritis. Infect. Immun..

[30] Kang M., Ko Y.-P., Liang X., Ross C.L., Liu Q., Murray B.E., Höök M. (2013). Collagen-binding Microbial Surface Components Recognizing Adhesive Matrix Molecule (MSCRAMM) of Gram-positive Bacteria Inhibit Complement Activation via the Classical Pathway. J. Biol. Chem..

[31] Falugi F., Kim H.K., Missiakas D.M., Schneewind O. (2013). Role of Protein A in the Evasion of Host Adaptive Immune Responses by Staphylococcus aureus. mBio.

[32] O’Seaghdha M., Van Schooten C.J., Kerrigan S.W., Emsley J., Silverman G.J., Cox D., Lenting P.J., Foster T.J. (2006). Staphylococcus aureusprotein A binding to von Willebrand factor A1 domain is mediated by conserved IgG binding regions. FEBS J..

[33] Gómez M.I., O’Seaghdha M., Magargee M., Foster T.J., Prince A.S. (2006). Staphylococcus aureus Protein A Activates TNFR1 Signaling through Conserved IgG Binding Domains. J. Biol. Chem..

[34] Viela F., Prystopiuk V., Leprince A., Mahillon J., Speziale P., Pietrocola G., Dufrêne Y.F. (2019). Binding ofStaphylococcus aureusProtein A to von Willebrand Factor Is Regulated by Mechanical Force. mBio.

[35] Yang Y.-H., Jiang Y.-L., Zhang J., Wang L., Bai X.-H., Zhang S.-J., Ren Y.-M., Li N., Zhang Y.-H., Zhang Z. (2014). Structural Insights into SraP-Mediated Staphylococcus aureus Adhesion to Host Cells. PLoS Pathog..

[36] Kukita K., Kawada-Matsuo M., Oho T., Nagatomo M., Oogai Y., Hashimoto M., Suda Y., Tanaka T., Komatsuzawa H. (2013). Staphylococcus aureus SasA Is Responsible for Binding to the Salivary Agglutinin gp340, Derived from Human Saliva. Infect. Immun..

[37] Torres V.J., Pishchany G., Humayun M., Schneewind O., Skaar E.P. (2006). Staphylococcus aureus IsdB Is a Hemoglobin Receptor Required for Heme Iron Utilization. J. Bacteriol..

[38] Pietrocola G., Pellegrini A., Alfeo M.J., Marchese L., Foster T.J., Speziale P. (2020). The iron-regulated surface determinant B (IsdB) protein from Staphylococcus aureus acts as a receptor for the host protein vitronectin. J. Biol. Chem..

[39] Ponnuraj K., Bowden M., Davis S., Gurusiddappa S., Moore D., Choe D., Xu Y., Hook M., Narayana S.V. (2003). A “dock, lock, and latch” Structural Model for a Staphylococcal Adhesin Binding to Fibrinogen. Cell.

[40] Rohde H., Burdelski C., Bartscht K., Hussain M., Buck F., Horstkotte M.A., Knobloch J.K.-M., Heilmann C., Herrmann M., Mack D. (2005). Induction ofStaphylococcus epidermidisbiofilm formation via proteolytic processing of the accumulation-associated protein by staphylococcal and host proteases. Mol. Microbiol..

[41] Paharik A.E., Kotasinska M., Both A., Hoang T.-M.N., Büttner H., Roy P., Fey P.D., Horswill A.R., Rohde H. (2017). The metalloprotease SepA governs processing of accumulation-associated protein and shapes intercellular adhesive surface properties inStaphylococcus epidermidis. Mol. Microbiol..

[42] Brusselmans K., Timmermans L., Van de Sande T., Van Veldhoven P.P., Guan G., Shechter I., Claessens F., Verhoeven G., Swinnen J.V. (2007). Squalene Synthase, a Determinant of Raft-associated Cholesterol and Modulator of Cancer Cell Proliferation. J. Biol. Chem..

[43] Shahrooei M., Hira V., Stijlemans B., Merckx R., Hermans P.W.M., Van Eldere J. (2009). Inhibition of Staphylococcus epidermidis Biofilm Formation by Rabbit Polyclonal Antibodies against the SesC Protein. Infect. Immun..

[44] Khodaparast L., Khodaparast L., Van Mellaert L., Shahrooei M., Van Ranst M., Van Eldere J. (2016). sesC as a genetic marker for easy identification of Staphylococcus epidermidis from other isolates. Infect. Genet. Evol..

[45] Bartual S.G., Alcorlo M., Martínez-Caballero S., Molina R., Hermoso J.A. (2018). Three-dimensional structures of Lipoproteins from Streptococcus pneumoniae and Staphylococcus aureus. Int. J. Med. Microbiol..

[46] Mariotti P., Malito E., Biancucci M., Surdo P.L., Mishra R.P.N., Nardi-Dei V., Savino S., Nissum M., Spraggon G., Grandi G. (2013). Structural and functional characterization of the Staphylococcus aureus virulence factor and vaccine candidate FhuD2. Biochem. J..

[47] Horsburgh M.J., Wharton S.J., Cox A.G., Ingham E., Peacock S., Foster S.J. (2002). MntR modulates expression of the PerR regulon and superoxide resistance in Staphylococcus aureus through control of manganese uptake. Mol. Microbiol..

[48] Tribelli P.M., Luqman A., Nguyen M., Madlung J., Fan S., Macek B., Sass P., Bitschar K., Schittek B., Kretschmer D. (2020). Staphylococcus aureus Lpl protein triggers human host cell invasion via activation of Hsp90 receptor. Cell. Microbiol..

[49] Liu Y., Liu F.-J., Guan Z.-C., Dong F.-T., Cheng J.-H., Gao Y.-P., Li D., Yan J., Liu C.-H., Han D.-P. (2018). The extracellular domain of Staphylococcus aureus LtaS binds insulin and induces insulin resistance during infection. Nat. Microbiol..

[50] Heilmann C., Thumm G., Chhatwal G.S., Hartleib J., Uekötter A., Peters G. (2003). Identification and characterization of a novel autolysin (Aae) with adhesive properties from Staphylococcus epidermidis. Microbiol..

[51] Bowden M.G., Visai L., Longshaw C.M., Holland K.T., Speziale P., Höök M. (2002). Is the GehD Lipase from Staphylococcus epidermidis a Collagen Binding Adhesin?. J. Biol. Chem..

[52] Christner M., Franke G.C., Schommer N.N., Wendt U., Wegert K., Pehle P., Kroll G., Schulze C., Buck F., Mack D. (2010). The giant extracellular matrix-binding protein ofStaphylococcus epidermidismediates biofilm accumulation and attachment to fibronectin. Mol. Microbiol..

[53] Büttner H., Perbandt M., Kohler T., Kikhney A., Wolters M., Christner M., Heise M., Wilde J., Weißelberg S., Both A. (2020). A Giant Extracellular Matrix Binding Protein of Staphylococcus epidermidis Binds Surface-Immobilized Fibronectin via a Novel Mechanism. mBio.

[54] Rooijakkers S.H.M., Ruyken M., Van Roon J., Van Kessel K.P.M., Van Strijp J.A.G., Van Wamel W.J.B. (2006). Early expression of SCIN and CHIPS drives instant immune evasion by Staphylococcus aureus. Cell. Microbiol..

[55] De Haas C.J., Veldkamp K.E., Peschel A., Weerkamp F., Van Wamel W.J., Heezius E.C., Poppelier M.J., Van Kessel K.P., Van Strijp J.A. (2004). Chemotaxis Inhibitory Protein of Staphylococcus aureus, a Bacterial Antiinflammatory Agent. J. Exp. Med..

[56] Postma B., Kleibeuker W., Poppelier M.J.J.G., Boonstra M., Van Kessel K.P.M., Van Strijp J.A.G., De Haas C.J.C. (2005). Residues 10–18 within the C5a Receptor N Terminus Compose a Binding Domain for Chemotaxis Inhibitory Protein of Staphylococcus aureus. J. Biol. Chem..

[57] Jongerius I., Köhl J., Pandey M.K., Ruyken M., Van Kessel K.P., Van Strijp J.A., Rooijakkers S.H. (2007). Staphylococcal complement evasion by various convertase-blocking molecules. J. Exp. Med..

[58] Ko Y.-P., Liang X., Smith C.W., Degen J.L., Höök M. (2011). Binding of Efb from Staphylococcus aureus to Fibrinogen Blocks Neutrophil Adherence*. J. Biol. Chem..

[59] Woehl J.L., Stapels D.A.C., Garcia B.L., Ramyar K.X., Keightley A., Ruyken M., Syriga M., Sfyroera G., Weber A.B., Zolkiewski M. (2014). The Extracellular Adherence Protein fromStaphylococcus aureusInhibits the Classical and Lectin Pathways of Complement by Blocking Formation of the C3 Proconvertase. J. Immunol..

[60] Stapels D.A., Ramyar K.X., Bischoff M., Köckritz-Blickwede M., Milder F.J., Ruyken M., Eisenbeis J., McWhorter W.J., Herrmann M., Kessel K.P. (2014). Staphylococcus aureus secretes a unique class of neutrophil serine protease inhibitors. Proc. Natl. Acad. Sci. USA.

[61] Panizzi P., Friedrich R., Fuentes-Prior P., Kroh H.K., Briggs J., Tans G., Bode W., Bock P.E. (2006). Novel Fluorescent Prothrombin Analogs as Probes of Staphylocoagulase-Prothrombin Interactions. J. Biol. Chem..

[62] Friedrich R., Panizzi P., Fuentes-Prior P., Richter K., Verhamme I., Anderson P.J., Kawabata S.-I., Huber R., Bode W., Bock P.E. (2003). Staphylocoagulase is a prototype for the mechanism of cofactor-induced zymogen activation. Nat. Cell Biol..

[63] Bjerketorp J., Nilsson M., Ljungh Å., Flock J.-I., Jacobsson K., Frykberg L. (2002). A novel von Willebrand factor binding protein expressed by Staphylococcus aureus a The GenBank accession number for the sequence reported in this paper is AY032850. Microbiology.

[64] Hendrix H., Lindhout T., Mertens K., Engels W., Hemker H.C. (1983). Activation of human prothrombin by stoichiometric levels of staphylocoagulase. J. Biol. Chem..

[65] Kroh H.K., Panizzi P., Bock P.E. (2009). Von Willebrand factor-binding protein is a hysteretic conformational activator of prothrombin. Proc. Natl. Acad. Sci. USA.

[66] Thomer L., Schneewind O., Missiakas D. (2013). Multiple Ligands of von Willebrand Factor-binding Protein (vWbp) Promote Staphylococcus aureus Clot Formation in Human Plasma. J. Biol. Chem..

[67] Thomas S., Liu W., Arora S., Ganesh V., Ko Y.-P., Höök M. (2019). The Complex Fibrinogen Interactions of the Staphylococcus aureus Coagulases. Front. Cell. Infect. Microbiol..

[68] Bjerketorp J., Jacobsson K., Frykberg L. (2004). The von Willebrand factor-binding protein (vWbp) of Staphylococcus aureus is a coagulase. FEMS Microbiol. Lett..

[69] Tam K., Torres V.J. (2019). Staphylococcus aureus Secreted Toxins and Extracellular Enzymes. Microbiol. Spectr..

[70] Caiazza N.C., O’Toole G.A. (2003). Alpha-Toxin Is Required for Biofilm Formation by Staphylococcus aureus. J. Bacteriol..

[71] Anderson M.J., Schaaf E., Breshears L.M., Wallis H.W., Johnson J.R., Tkaczyk C., Sellman B.R., Sun J., Peterson M.L. (2018). Alpha-Toxin Contributes to Biofilm Formation among Staphylococcus aureus Wound Isolates. Toxins.

[72] Scherr T.D., Hanke M.L., Huang O., James D.B.A., Horswill A.R., Bayles K.W., Fey P.D., Torres V.J., Kielian T. (2015). Staphylococcus aureus Biofilms Induce Macrophage Dysfunction Through Leukocidin AB and Alpha-Toxin. mBio.

[73] Estellés A., Woischnig A.-K., Liu K., Stephenson R., Lomongsod E., Nguyen D., Zhang J., Heidecker M., Yang Y., Simon R.J. (2016). A High-Affinity Native Human Antibody Disrupts Biofilm from Staphylococcus aureus Bacteria and Potentiates Antibiotic Efficacy in a Mouse Implant Infection Model. Antimicrob. Agents Chemother..

[74] Xiong Y.Q., Estellés A., Li L., Abdelhady W., Gonzales R., Bayer A.S., Tenorio E., Leighton A., Ryser S., Kauvar L.M. (2017). A Human Biofilm-Disrupting Monoclonal Antibody Potentiates Antibiotic Efficacy in Rodent Models of both Staphylococcus aureus and Acinetobacter baumannii Infections. Antimicrob. Agents Chemother..

[75] Goodman S.D., Obergfell K.P., Jurcisek J.A., Novotny L.A., Downey J.S., Ayala E.A., Tjokro N.O., Li B., Justice S.S., Bakaletz L.O. (2011). Biofilms can be dispersed by focusing the immune system on a common family of bacterial nucleoid-associated proteins. Mucosal Immunol..

[76] Kuby J., Schroeder H.W. (1994). Immunoglobulins: Structure and Functions. Immunology.

[77] Janeway C.A., Travers P., Walport M., Shlomchik M.J. (2001). The generation of lymphocyte antigen receptors. Immuno-Biology.

[78] Moldenhauer G., Dübel S. (2010). Selecting strategies I: Monoclonal antibodies. Handbook of Therapeutic Antibodies.

[79] Hall A.E., Domanski P.J., Patel P.R., Vernachio J.H., Syribeys P.J., Gorovits E.L., Johnson M.A., Ross J.M., Hutchins J.T., Patti J.M. (2003). Characterization of a Protective Monoclonal AntibodyRecognizing Staphylococcus aureus MSCRAMM ProteinClumping FactorA. Infect. Immun..

[80] Patti J.M. (2004). A humanized monoclonal antibody targeting Staphylococcus aureus. Vaccine.

[81] Weems J.J., Steinberg J.P., Filler S., Baddley J.W., Corey G.R., Sampathkumar P., Winston L., John J.F., Kubin C.J., Talwani R. (2006). Phase II, Randomized, Double-Blind, Multicenter Study Comparing the Safety and Pharmacokinetics of Tefibazumab to Placebo for Treatment of Staphylococcus aureus Bacteremia. Antimicrob. Agents Chemother..

[82] Schaffer A.C., Solinga R.M., Cocchiaro J., Portoles M., Kiser K.B., Risley A., Randall S.M., Valtulina V., Speziale P., Walsh E. (2006). Immunization with Staphylococcus aureus Clumping Factor B, a Major Determinant in Nasal Carriage, Reduces Nasal Colonization in a Murine Model. Infect. Immun..

[83] Visai L., Xu Y., Casolini F., Rindi S., Höök M., Speziale P. (2000). Monoclonal Antibodies to CNA, a Collagen-binding Microbial Surface Component Recognizing Adhesive Matrix Molecules, DetachStaphylococcus aureus from a Collagen Substrate. J. Biol. Chem..

[84] Herman-Bausier P., Valotteau C., Pietrocola G., Rindi S., Alsteens D., Foster T.J., Speziale P., Dufrene Y.F. (2016). Mechanical Strength and Inhibition of the Staphylococcus aureus Collagen-Binding Protein Cna. mBio.

[85] Kim H.K., Emolo C., DeDent A.C., Falugi F., Missiakas D.M., Schneewind O. (2012). Protein A-Specific Monoclonal Antibodies and Prevention of Staphylococcus aureus Disease in Mice. Infect. Immun..

[86] Kim H.K., Cheng A.G., Kim H.-Y., Missiakas D.M., Schneewind O. (2010). Nontoxigenic protein A vaccine for methicillin-resistant Staphylococcus aureus infections in mice. J. Exp. Med..

[87] Chen X., Sun Y., Missiakas D., Schneewind O. (2019). Staphylococcus aureus Decolonization of Mice With Monoclonal Antibody Neutralizing Protein A. J. Infect. Dis..

[88] Thammavongsa V., Rauch S., Kim H.K., Missiakas D.M., Schneewind O. (2015). Protein A-neutralizing monoclonal antibody protects neonatal mice against Staphylococcus aureus. Vaccine.

[89] Varshney A.K., Kuzmicheva G.A., Lin J., Sunley K.M., Bowling R.A., Kwan T.-Y., Mays H.R., Rambhadran A., Zhang Y., Martin R.L. (2018). A natural human monoclonal antibody targeting Staphylococcus Protein A protects against Staphylococcus aureus bacteremia. PLoS ONE.

[90] Zhou T.-T., Yue Y., Zheng F., Liang X.-D., Cao Q.-X., Wang Y.-W., Zhu J. (2019). Monoclonal antibody against l-lectin module of SraP blocks adhesion and protects mice against Staphylococcus aureus challenge. J. Microbiol. Immunol. Infect..

[91] Thomer L., Emolo C., Thammavongsa V., Kim H.K., McAdow M.E., Yu W., Kieffer M., Schneewind O., Missiakas D. (2016). Antibodies against a secreted product of Staphylococcus aureus trigger phagocytic killing. J. Exp. Med..

[92] Park J., Jagasia R., Kaufmann G.F., Mathison J.C., Ruiz D.I., Moss J.A., Meijler M.M., Ulevitch R.J., Janda K.D. (2007). Infection Control by Antibody Disruption of Bacterial Quorum Sensing Signaling. Chem. Biol..

[93] Kirchdoerfer R.N., Garner A.L., Flack C.E., Mee J.M., Horswill A.R., Janda K.D., Kaufmann G.F., Wilson I.A. (2011). Structural Basis for Ligand Recognition and Discrimination of a Quorum-quenching Antibody. J. Biol. Chem..

[94] Oganesyan V., Peng L., Damschroder M.M., Cheng L., Sadowska A., Tkaczyk C., Sellman B.R., Wu H., Dall’Acqua W.F. (2014). Mechanisms of Neutralization of a Human Anti-α-toxin Antibody. J. Biol. Chem..

[95] Hua L., Hilliard J.J., Shi Y., Tkaczyk C., Cheng L.I., Yu X., Datta V., Ren S., Feng H., Zinsou R. (2013). Assessment of an Anti-Alpha-Toxin Monoclonal Antibody for Prevention and Treatment of Staphylococcus aureus-Induced Pneumonia. Antimicrob. Agents Chemother..

[96] Yu X.-Q., Robbie G.J., Wu Y., Esser M.T., Jensen K., Schwartz H.I., Bellamy T., Hernandez-Illas M., Jafri H.S. (2017). Safety, Tolerability, and Pharmacokinetics of MEDI4893, an Investigational, Extended-Half-Life, Anti-Staphylococcus aureus Alpha-Toxin Human Monoclonal Antibody, in Healthy Adults. Antimicrob. Agents Chemother..

[97] Ruzin A., Wu Y., Yu L., Yu X.-Q., Tabor D.E., Mok H., Tkaczyk C., Jensen K., Bellamy T., Roskos L. (2018). Charac-terisation of anti-alpha toxin antibody levels and colonisation status after administration of an investigational human monoclonal antibody, MEDI4893, against Staphylococcus aureus alpha toxin. Clin. Transl. Immunol..

[98] Aguilar J.L., Varshney A.K., Pechuan X., Dutta K., Nosanchuk J.D., Fries B.C. (2016). Monoclonal antibodies protect from Staphylococcal Enterotoxin K (SEK) induced toxic shock and sepsis by USA300Staphylococcus aureus. Virulence.

[99] Hall A.E., Patel P.R., Domanski P.J., Prater B.D., Gorovits E.L., Syribeys P.J., Vernachio J.H., Patti J.M., Hutchins J.T. (2007). A Panel of Monoclonal Antibodies Recognizing theStaphylococcus epidermidisFibrinogen-BindingMSCRAMM SdrG. Hybridoma.

[100] Brown M., Kowalski R., Zorman J., Wang X.-M., Towne V., Zhao Q., Secore S., Finnefrock A.C., Ebert T., Pancari G. (2009). Selection and Characterization of Murine Monoclonal Antibodies to Staphylococcus aureus Iron-Regulated Surface Determinant B with Functional Activity In Vitro and In Vivo. Clin. Vaccine Immunol..

[101] Chen X., Shi M., Tong X., Kim H.K., Wang L.-X., Schneewind O., Missiakas D. (2020). Glycosylation-dependent opsonophagocytic activity of staphylococcal protein A antibodies. Proc. Natl. Acad. Sci. USA.

[102] Huynh T., Stecher M., McKinnon J., Jung N., Rupp M.E. (2016). Safety and Tolerability of 514G3, a True Human Anti-Protein A Monoclonal Antibody for the Treatment of S. aureus Bacteremia. Open Forum Infect. Dis..

[103] Yang Y., Qian M., Yi S., Liu S., Li B., Yu R., Guo Q., Zhang X., Yu C., Li J. (2016). Monoclonal Antibody Targeting Staphylococcus aureus Surface Protein A (SasA) Protect Against Staphylococcus aureus Sepsis and Peritonitis in Mice. PLoS ONE.

[104] Anderson A.S., Scully I.L., Timofeyeva Y., Murphy E., McNeil L.K., Mininni T., Nuñez L., Carriere M., Singer C., Dilts D.A. (2012). Staphylococcus aureus Manganese Transport Protein C Is a Highly Conserved Cell Surface Protein That Elicits Protective Immunity Against S. aureus and Staphylococcus epidermidis. J. Infect. Dis..

[105] Burnie J.P., Matthews R.C., Carter T., Beaulieu E., Donohoe M., Chapman C., Williamson P., Hodgetts S.J. (2000). Identification of an Immunodominant ABC Transporter in Methicillin-Resistant Staphylococcus aureusInfections. Infect. Immun..

[106] Otto M. (2010). Novel targeted immunotherapy approaches for staphylococcal infection. Expert Opin. Biol. Ther..

[107] Baker M. (2006). Anti-infective antibodies: Finding the path forward. Nat. Biotechnol..

[108] Georgoutsou-Spyridonos M., Ricklin D., Pratsinis H., Perivolioti E., Pirmettis I., Garcia B.L., Geisbrecht B.V., Foukas P.G., Lambris J.D., Mastellos D.C. (2015). Attenuation ofStaphylococcus aureus–Induced Bacteremia by Human Mini-Antibodies Targeting the Complement Inhibitory Protein Efb. J. Immunol..

[109] Haas P.-J., De Haas C.J.C., Kleibeuker W., Poppelier M.J.J.G., Van Kessel K.P.M., Kruijtzer J.A.W., Liskamp R.M.J., Van Strijp J.A.G. (2004). N-Terminal Residues of the Chemotaxis Inhibitory Protein of Staphylococcus aureus Are Essential for Blocking Formylated Peptide Receptor but Not C5a Receptor. J. Immunol..

[110] Hoekstra H., Pastrana F.R., Bonarius H.P.J., Van Kessel K.P.M., Elsinga G.S., Kooi N., Groen H., Van Dijl J.M., Buist G. (2018). A human monoclonal antibody that specifically binds and inhibits the staphylococcal complement inhibitor protein SCIN. Virulence.

[111] Rouha H., Badarau A., Visram Z.C., Battles M.B., Prinz B., Magyarics Z., Nagy G., Mirkina I., Stulik L., Zerbs M. (2015). Five birds, one stone: Neutralization of α-hemolysin and 4 bi-component leukocidins of Staphylococcus aureus with a single human monoclonal antibody. mAbs.

[112] Stulik L., Rouha H., Labrousse D., Visram Z.C., Badarau A., Maierhofer B., Groß K., Weber S., Kramarić M.D., Glojnarić I. (2019). Preventing lung pathology and mortality in rabbit Staphylococcus aureus pneumonia models with cytotoxin-neutralizing monoclonal IgGs penetrating the epithelial lining fluid. Sci. Rep..

[113] Varshney A.K., Wang X., MacIntyre J., Zollner R.S., Kelleher K., Kovalenko O.V., Pechuan X., Byrne F.R., Fries B.C. (2014). Humanized Staphylococcal Enterotoxin B (SEB)–Specific Monoclonal Antibodies Protect From SEB Intoxication and Staphylococcus aureus Infections Alone or as Adjunctive Therapy With Vancomycin. J. Infect. Dis..

[114] Karau M.J., Tilahun M.E., Krogman A., Osborne B.A., Goldsby R.A., David C.S., Mandrekar J.N., Patel R., Rajagopalan G. (2017). Passive therapy with humanized anti-staphylococcal enterotoxin B antibodies attenuates systemic inflammatory response and protects from lethal pneumonia caused by staphylococcal enterotoxin B-producingStaphylococcus aureus. Virulence.

[115] Rukkawattanakul T., Sookrung N., Seesuay W., Onlamoon N., Diraphat P., Chaicumpa W., Indrawattana N. (2017). Human scFvs That Counteract Bioactivities of Staphylococcus aureus TSST-1. Toxins.

[116] Sun D., Accavitti M.A., Bryers J.D. (2005). Inhibition of Biofilm Formation by Monoclonal Antibodies against Staphylococcus epidermidis RP62A Accumulation-Associated Protein. Clin. Diagn. Lab. Immunol..

[117] Ortines R.V., Liu H., Cheng L.I., Cohen T.S., Lawlor H., Gami A., Wang Y., Dillen C.A., Archer N.K., Miller R.J. (2018). Neutralizing Alpha-Toxin Accelerates Healing of Staphylococcus aureus -Infected Wounds in Nondiabetic and Diabetic Mice. Antimicrob. Agents Chemother..

[118] Wang Y., Cheng L.I., Helfer D.R., Ashbaugh A.G., Miller R.J., Tzomides A.J., Thompson J.M., Ortines R.V., Tsai A.S., Liu H. (2017). Mouse model of hematogenous implant-relatedStaphylococcus aureusbiofilm infection reveals therapeutic targets. Proc. Natl. Acad. Sci. USA.

[119] Lehar S.M., Pillow T., Xu M., Staben L., Kajihara K.K., Vandlen R., DePalatis L., Raab H., Hazenbos W.L., Morisaki J.H. (2015). Novel antibody–antibiotic conjugate eliminates intracellular *S. aureus*. Nature.

[120] Mariathasan S., Tan M.-W. (2017). Antibody–Antibiotic Conjugates: A Novel Therapeutic Platform against Bacterial Infections. Trends Mol. Med..

[121] Bispo M., Anaya-Sanchez A., Suhani S., Raineri E.J.M., López-Álvarez M., Heuker M., Szymański W., Pastrana F.R., Buist G., Horswill A.R. (2020). Fighting Staphylococcus aureus infections with light and photoimmunoconjugates. JCI Insight.

[122] Millenbaugh N.J., Baskin J.B., DeSilva M.N., Elliott W.R., Glickman R.D. (2015). Photothermal killing of Staphylococcus aureus using antibody-targeted gold nanoparticles. Int. J. Nanomed..

[123] Vor L., Dijk B., Kessel K.P.M., Kavanaugh J.S., Haas C.J.C., Aerts P.C., Viveen M.C., Boel E.C.H., Fluit A.D.C., Kwiecinski J.M. (2021). Human monoclonal antibodies against Staphylococcus aureus surface antigens recognize in vitro biofilm and in vivo implant infections. Biorxiv Microbiol..

[124] Swerlick R.A., Cunningham M.W., Hall N.K. (1986). Monoclonal Antibodies Cross-Reactive with Group A Streptococci and Normal and Psoriatic Human Skin. J. Investig. Dermatol..

[125] Descotes J. (2009). Immunotoxicity of monoclonal antibodies. mAbs.

